# Metagenome Sequencing of the Prokaryotic Microbiota of the Hypersaline and Meromictic Soap Lake, Washington

**DOI:** 10.1128/genomeA.01212-13

**Published:** 2014-01-23

**Authors:** Erik R. Hawley, Matthias Hess

**Affiliations:** a Washington State University Tri-Cities, Richland, Washington, USA; b DOE Joint Genome Institute, Walnut Creek, California, USA; c Washington State University, Pullman, Washington, USA; d Pacific Northwest National Laboratory, Chemical & Biological Process Development Group, Richland, Washington, USA

## Abstract

Soap Lake is a small saline lake in central eastern Washington that is sharply stratified into two layers. In addition to being highly alkaline (~pH 10), Soap Lake also contains high concentrations of sulfide. Here, we report the community profile of the prokaryotic microbiota associated with Soap Lake surface water.

## GENOME ANNOUNCEMENT

Soda lakes and deserts are the most stable alkaline ecosystems that occur naturally on our planet (1). The ability of sodium carbonates to maintain stable alkaline pH values between 9.5 and 10.5 makes them ideal habitats for alkaliphilic microorganisms (2). The primary production in these lakes results mostly from the metabolic activities of purple sulfur bacteria and *Cyanobacteria* (3), and the daily primary production of these ecosystems exceeds in many cases the primary production measured for eutrophic lakes (4). Cultivation-independent approaches, such as DNA sequencing, are valuable tools to obtain a better understanding of the microbial communities of these extreme environments, which is a crucial step toward elucidating the factors that underlie the exceptional productivity of these ecosystems. Here, we report the metagenome, as determined by 16S rRNA amplicon sequencing, of Soap Lake surface water. Soap Lake is a small soda lake in east central Washington with a closed basin system that is permanently stratified due to the highly saline (141 g/liter) monimolimnion and the less-saline (18 g/liter) mixolimnion (5).

A sample was collected on 1 September 2012 (global positioning system [GPS] coordinates 47.421223, -119.495255) using a sterile 50-ml sampling tube. The water temperature and pH were 20°C and 10, respectively. DNA was extracted using the FastDNA SPIN kit for soil from MP Biomedicals, according to the manufacturer’s protocol. The V6 to V8 region of the bacterial 16S rRNA gene was amplified using the primer set 926f/1392r and sequenced using the Roche 454 FLX+ platform (Research and Testing Laboratory, Lubbock, TX). The raw pyrosequence reads were quality filtered and analyzed using QIIME version 1.7.0 (6). Prior to filtering, the sequencing primers and barcodes were removed, allowing 1.5 mismatches to the barcode and 2 mismatches to the primer. The sequences were removed from analysis if they contained homopolymers >6 bp, were <200 bp in length, contained a quality score <25, or if they were found to be chimeric. The sequences were clustered into operational taxonomic units (OTUs) at the 97% sequence identity level using UCLUST (7), and the most abundant sequence of each OTU was chosen as a representative. The OTU representative sequences were aligned using PyNAST (8) and then filtered to remove common gaps. The reference sequences of each OTU were taxonomically classified using the RDP Classifier (9) with an 80% confidence rating against the Greengenes database (10).

A total of 2,702 high-quality sequences, representing 172 distinct OTUs and 13 phyla, were obtained, and a Good’s coverage of 0.98 was calculated. The five most abundant phyla were *Firmicutes* (45.4%), *Proteobacteria* (15.4%)*, Tenericutes* (10.5%)*, Actinobacteria* (10.2%), and *Bacteroidetes* (5.1%). *Cyanobacteria* contributed 3.2% of the sequence reads. Interestingly, 80.3% of the phylogenomic data were recruited by 18 OTUs, and 41.8% were by a single OTU assigned to an uncultured microorganism classified as a member of the *Firmicutes*. This data set reveals a relatively low diversity of microorganisms capable of coping with the extreme conditions of Soap Lake.

### Nucleotide sequence accession number.

The DNA sequences from this metagenome project have been deposited in the NCBI Short Read Archive under the accession no. SRP033727.
